# Modeling halotropism: a key role for root tip architecture and reflux loop remodeling in redistributing auxin

**DOI:** 10.1242/dev.135111

**Published:** 2016-09-15

**Authors:** Thea van den Berg, Ruud A. Korver, Christa Testerink, Kirsten H. W. J. ten Tusscher

**Affiliations:** 1 Theoretical Biology, Department of Biology, Utrecht University, 3584 CH Utrecht, The Netherlands; 2 Plant Cell Biology, Swammerdam Institute for Life Sciences, University of Amsterdam, 1090 GE Amsterdam, The Netherlands

**Keywords:** Plant development, Root tropism, Auxin transport, Modeling, Computer simulation

## Abstract

A key characteristic of plant development is its plasticity in response to various and dynamically changing environmental conditions. Tropisms contribute to this flexibility by allowing plant organs to grow from or towards environmental cues. Halotropism is a recently described tropism in which plant roots bend away from salt. During halotropism, as in most other tropisms, directional growth is generated through an asymmetric auxin distribution that generates differences in growth rate and hence induces bending. Here, we develop a detailed model of auxin transport in the *Arabidopsis* root tip and combine this with experiments to investigate the processes generating auxin asymmetry during halotropism. Our model points to the key role of root tip architecture in allowing the decrease in PIN2 at the salt-exposed side of the root to result in a re-routing of auxin to the opposite side. In addition, our model demonstrates how feedback of auxin on the auxin transporter AUX1 amplifies this auxin asymmetry, while a salt-induced transient increase in PIN1 levels increases the speed at which this occurs. Using AUX1-GFP imaging and *pin1* mutants, we experimentally confirmed these model predictions, thus expanding our knowledge of the cellular basis of halotropism.

## Abstract (precis)

**Summary:** During halotropism, root tip architecture allows for a decrease in PIN2 at the salt-exposed side of the root, resulting in re-routing of auxin to the opposite side; feedback on AUX1 amplifies this auxin asymmetry.

## INTRODUCTION

Plant development is characterized by a large degree of flexibility, termed developmental or phenotypic plasticity. As a result of their sessile nature, plants have very limited influence over the environmental conditions they find themselves in. As a consequence, plants had to evolve the capacity to survive in different environments as well as dynamically changing environmental conditions by making their developmental programs dependent on environmental signals. The resulting phenotypic plasticity enables plants to flexibly adjust to their environmental conditions. This developmental plasticity may influence overall architecture, for example, the layout of the root system, by influencing meristem size and hence the rate of root growth (Aquea et al., 2012; Chapman et al., 2011; Jain et al., 2007; Liu et al., 2015; West et al., 2004), as well as the number of developing or outgrowing lateral roots (Jain et al., 2007). Another, perhaps more subtle, adaptation of plant development involves the directional growth of plant organs towards or away from a perceived stimulus, such as the gravity vector (Abas et al., 2006; Sukumar et al., 2009), light (Wan et al., 2012; Laxmi et al., 2008; Sassi et al., 2012) or nutrients (Niu et al., 2015). Recently, we described a new tropism, called halotropism, that entails the directional growth of plant roots away from salt (Galvan-Ampudia et al., 2013).

At the base of most plant tropisms lies an asymmetric distribution of the plant hormone auxin (Went, 1974) that generates asymmetric growth rates and thus causes bending. Auxin patterns are strongly determined by auxin transport. Auxin can enter cells both via passive diffusion as well as active transport mediated by the AUX/LAX family of importers (Rutschow et al., 2014; Swarup et al., 2008; Bennett et al., 1996). Auxin importers have a tissue-specific expression pattern (Bennett et al., 1996; Péret et al., 2012; Swarup et al., 2001, 2005) leading to the preferential retention of auxin in particular tissues. Auxin can only leave cells via active transport, a process that is dominated by the PIN family of exporters (Friml et al., 2003; Petrášek et al., 2006; Wisniewska et al., 2006; Paponov et al., 2005). In addition to having tissue-specific expression domains, PIN proteins have a tissue and PIN-type-specific polar membrane pattern, leading to directional auxin transport fluxes (Friml et al., 2002a, 2003; Benková et al., 2003). In plant roots, a reverse fountain PIN pattern generates a symmetrical auxin gradient with a maximum close to the root tip (Blilou et al., 2005; Grieneisen et al., 2007). During root tropisms, bending is caused by an asymmetric elevation of auxin in the expansion zone that causes an asymmetric repression of expansion rates (Scheitz et al., 2013; Thimann, 1936).

The best studied tropism in plant roots is gravitropism. Upon a gravitropic stimulus, columella cell statoliths sediment onto the downward oriented membrane face (Eshel and Beeckman, 2013). Via an unidentified mechanism, this causes the polarization of the normally apolar PIN3 and PIN7 proteins onto the downward membrane (Friml et al., 2002b). As a consequence, most auxin arriving in the root tip now becomes transported towards the downward side. The auxin dependence of PIN2 membrane levels (Chen et al., 1998; Abas et al., 2006) and AUX1 gene expression levels (Laskowski et al., 2006, 2008) enable these transporters to amplify this initial auxin asymmetry and transport part of the excess auxin towards the expansion zone where it can affect expansion rates and induce root bending (Chen et al., 1998; Abas et al., 2006; Luschnig et al., 1998; Müller et al., 1998; Bennett et al., 1996; Swarup et al., 2001). Thus, while PIN3 and PIN7 appear to have a primary, asymmetry-inducing role, PIN2 and AUX1 appear to have a secondary, amplifying and transducing role (Eshel and Beeckman, 2013).

Intriguingly, we reported in an earlier study that the salt-induced auxin asymmetry causing halotropic root bending co-occurred with a reduction of epidermal PIN2 at the salt-exposed side of the root. No asymmetries in other PINs were reported. These data imply that PIN2, which has a mostly secondary role in gravitropism (Abas et al., 2006; Wan et al., 2012; Kleine-Vehn et al., 2008), plays a primary, asymmetry-generating role in halotropism. The gravitropism induced polarity switch in PIN3 and PIN7 in the root tip, where all auxin fluxes converge, biases auxin transport in one direction. This causes an auxin increase at one side of the root and an auxin decrease at the opposite side as two sides of the same coin. In contrast, although it can be easily understood that the halotropism-induced reduction of epidermal PIN2 will lead to a decrease of auxin at the salt-exposed side, it is far less trivial to see how this should lead to a concomitant increase in auxin at the opposite side. As a consequence, currently two alternative scenarios remain possible. In the first, PIN2 is the sole auxin asymmetry generator, and the reduced transport of auxin at one side of the reflux loop somehow leads to a translocation of this auxin towards the opposite side of the reflux loop. Alternatively, another unidentified auxin asymmetry-generating source may be present under halotropism.

In the current study, we use a detailed model of auxin transport in the *Arabidopsis* root tip to investigate whether the measured changes in PIN2 are necessary and sufficient to explain the auxin asymmetries observed under halotropism. We combine our modeling with experiments aimed at unraveling the potential role of salt-induced changes in other PIN proteins in generating or amplifying auxin asymmetry, as well as to confirm predictions generated by our model. Our computer simulations reveal the crucial importance of taking into account a realistic wedge-shaped root tip architecture for studying root tropisms. In absence of this realistic architecture, a PIN2 reduction at the salt-exposed side fails to induce any auxin increase at the opposite side, while in its presence, a modest auxin increase is automatically induced. We show that this increase was enhanced substantially when taking into account the auxin dependence of AUX1 (Bennett et al., 1996) and PIN2 (Paciorek et al., 2005; Whitford et al., 2012; Baster et al., 2012). Furthermore, our model predicts that underlying this enhanced auxin asymmetry is an asymmetry in AUX1 and PIN2 patterns. We experimentally validate this prediction for AUX1, demonstrating that exposure to a salt gradient results in an elevation of AUX1 levels on the non-salt-exposed versus salt-exposed side. In addition, we experimentally demonstrate that exposure to a salt gradient induces a transient, symmetric upregulation of PIN1. Incorporating this in our model significantly amplifies the auxin asymmetry arising in the early phases of halotropism, thus speeding up the halotropic response. Finally, we experimentally validated this role of PIN1 in root halotropism, by showing that *pin1* mutants exhibit a delayed halotropic response.

Our study suggests that the observed changes in PIN2 are responsible for the primary generation of auxin asymmetry. This asymmetry is subsequently further enhanced by the feedback of auxin on PIN2 itself and AUX1, and effectively sped up by a transient upregulation of PIN1. Together, this provides the necessary and sufficient conditions for generating an auxin asymmetry capable of inducing effective root bending.

## RESULTS

### Halotropic auxin asymmetry

To be able to judge whether the auxin asymmetries occurring in our simulations are sufficient to explain halotropic root bending, we first need to establish the amount of auxin asymmetry actually occurring during halotropism. For root tropisms, it is well known that auxin elevation leads to repression of cell expansion rates (Mullen et al., 1998; Band et al., 2012). However, it is less clear whether the concomitant decrease in auxin at the opposite side of the root contributes to growth rate asymmetry and bending by stimulating growth rate. Thus, we take a conservative approach, assuming that tropic bending is only caused by auxin elevation and growth inhibition.

In an earlier study (Galvan-Ampudia et al., 2013), we quantified the changes in DR5 and DII-Venus auxin reporter under halotropism. A ∼20% reduction in DR5 and a ∼20% increase in DII-Venus was found at the salt-exposed side, and a ∼20% increase in DR5 and ∼10% decrease in DII-Venus at the non-salt-exposed side. In an earlier study by Band et al. (2012), it was shown that during gravitropism a ∼30% decrease in DII-Venus occurred on the lower side of the root and that this corresponds to an ∼100% increase in auxin levels. Extrapolating these data, it was approximated that the change in DII-Venus observed during halotropism corresponds to a ∼30-40% increase in auxin levels.

### Root tip architecture

**Fig. 1.**
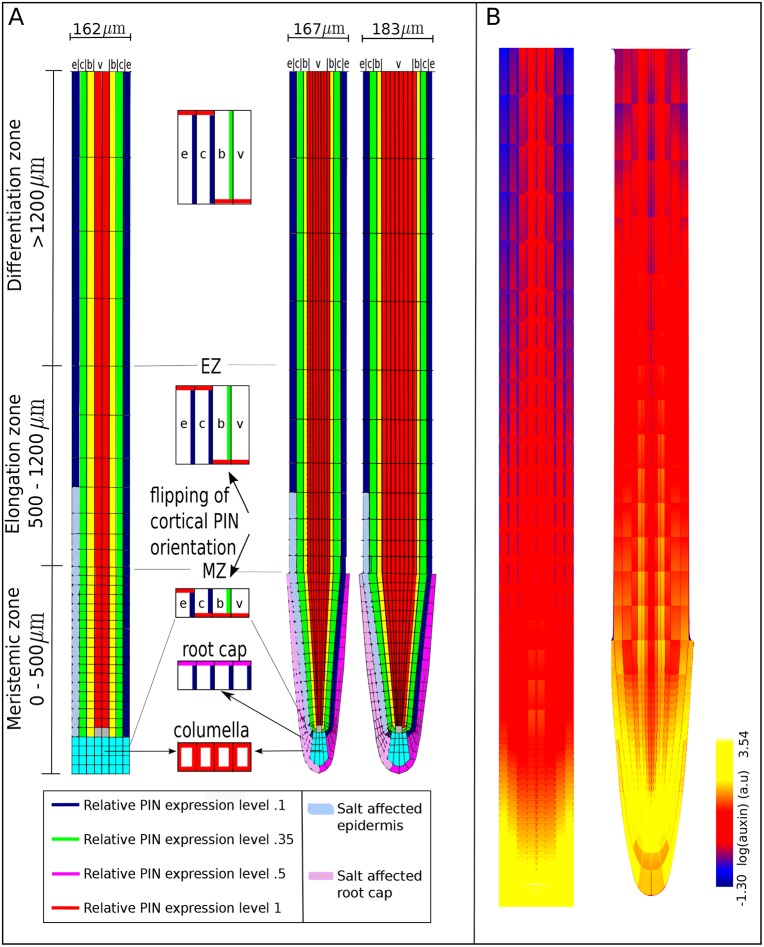
**Overview of model tissue layout.** (A) Layout of cell types, root zones and PIN polarity pattern in the baseline (left) and extended (middle) root models and a variant of the extended root model with larger left-right distances (right). Quiescent center (gray), columella (cyan), root cap (pink), epidermis (e, blue), cortex (c, green), border or endodermal cells (b, yellow) and vasculature (v, red). The root is divided into three zones, from bottom to top: meristem (MZ), elongation (EZ) and differentiation zone (DZ). Insets in the middle show the predefined PIN polarity pattern as present on the left side of the root in the different root zones (right side is a mirror image of the left side). An imposed salt gradient is assumed to influence PIN2 levels in lower left epidermal cells (light blue) and root cap cells (light pink). (B) Steady state non-salt stressed auxin pattern in the baseline model (left) and extended model (right).

The key question of this study is whether and how a reduction of epidermal PIN2 at the salt-stressed side can cause a rerouting of auxin to the non-salt-exposed side of the root. We hypothesize that root tip architecture plays a key role in this process. To investigate this, we developed three alternative root tip architectures. In the first, the baseline model, a highly simplified rectangular representation was used, similar to previous studies (Grieneisen et al., 2007; Laskowski et al., 2008; Mironova et al., 2010; Tian et al., 2014; Mähönen et al., 2014) (Fig. 1A, left). In the second, extended model, a realistic wedge-shaped root tip architecture containing root cap tissue was applied (Fig. 1A, middle). This architecture somewhat resembles the root tip model used in Cruz-Ramírez et al. (2012). Differences are the narrower root tip architecture, the stronger curvature and resulting smaller left-right distances close to the root tip, and the decrease in number of vascular cell files close to the root tip in our model, that we believe more realistically represent *Arabidopsis* root tip topology. The third architecture (Fig. 1A, right) is a variant of the second, in which vascular cells and outer columella cells are increased in width. It should be noted that during root aging, typically all tissues increase in width (Zhu et al., 1998). We are not modeling root aging here, rather, we increased the width of only the internal tissues to specifically investigate the impact of left-right distances between epidermal and lateral root cap tissues for auxin rerouting. For further details we refer to the Materials and Methods section.

Note that while in the baseline model only vascular tissue connects directly to the quiescent center (QC) and epidermal, cortical and endodermal cell files end on the columella, in the extended model, these tissues all end in a curvature directly on or near the QC. Furthermore, in the extended model the columella tiers are directly connected to either the epidermis or the lateral root cap. Since these tissues have a predominantly upward orientation of PIN polarity, all columella tiers are thus connected to shootward-transporting tissue files. By contrast, in the baseline model columella cells are connected both to upward transporting epidermal and downward transporting cortical and endodermal tissue. These differences can be expected to subtly affect properties of the auxin reflux loop. Indeed, in Fig. 1B, it can be observed that in the extended model auxin levels are reduced in the columella, and elevated in the epidermal and outer vascular cell files (right) compared with the baseline model (left).

### Salt-induced changes in PIN2

**Fig. 2.**
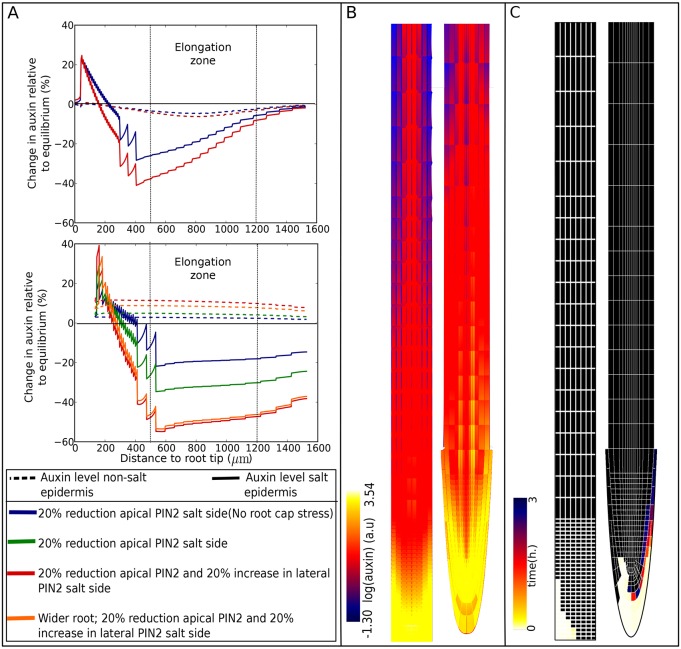
**Influence of salt-induced changes** **in PIN2 protein levels.** Salt stress was applied to the baseline and extended models, either by reducing only apical PIN2 levels or by also increasing lateral PIN2 levels. To investigate the impact of having a root cap, in the extended model, the reduction in apical PIN2 levels was applied only to epidermal cells, or to epidermal and root cap cells. To investigate the impact of distances between epidermal and root cap cells, results are also shown for an alternative realistic root tip architecture with increased left-right distances between epidermal and root cap cells, applying salt stress by decreasing apical and increasing lateral PIN2 levels. (A) Percentage changes in epidermal auxin levels on the salt-stressed and non-stressed side of the root relative to non-stressed conditions in the baseline (top) and extended (bottom) models. Location of the elongation zone is indicated. (B) Overall root tip auxin distributions for the scenario resulting in most auxin asymmetry in the baseline (left) and extended (right) models after 24 h of salt stress. (C) Auxin-rerouting maps for the scenario resulting in most auxin asymmetry in the baseline (left) and extended (right) model. For the baseline model, auxin rerouting was monitored by measuring the time at which at least a 1% increase in auxin levels occurred. For the extended model, a 10% auxin increase threshold was applied.

To monitor salt-gradient-induced auxin rerouting, we plot the percentage change in epidermal auxin levels (Fig. 2A), overall root pattern of auxin levels (Fig. 2B) and auxin rerouting (Fig. 2C). In the latter, we measure in each root cell whether auxin levels are elevated by at least 1% in case of the rectangular root topology, or at least 10% in case of the wedge-shaped root topology. We record the earliest time point at which such an elevation occurs and depict this time with a color code, thus generating a map of temporal auxin rerouting.

Halotropism simulations were started by applying a 20% reduction of apical PIN2 levels in epidermal cells on the salt stressed side of the baseline root model. This results in an asymmetric distribution of auxin (Fig. 2A, top and Fig. 2B, left). However, in contrast to experimental results where auxin decreases at the salt-stressed side and increases at the non-salt-stressed side, auxin decreases at both sides of the root. By mapping auxin rerouting, we see that halotropism results in a fast increase of auxin levels in the lowermost regions of the salt-exposed side (Fig. 2C, left). Clearly, the PIN2 reduction at the salt-exposed side results in a ‘traffic jam’, leading to auxin accumulation upstream of the transport blockage. No rerouting of auxin to the non-salt-exposed side occurs. Instead, the lower auxin levels at the non-exposed side imply that a reduction of overall reflux loop efficiency has occurred. Next, the same salt stress scenario was applied to our model extended with a realistic root tip architecture. Now, one can observe that besides a decrease of auxin at the salt-stressed side, auxin increases – albeit to a minor extent – at the non stressed side (Fig. 2A, bottom). Besides its modified root tip shape the extended model also contains root cap tissue. The rootcap has a similar PIN2 expression as the epidermis. It is therefore likely that the root cap PIN proteins are similarly affected by salt stress. When root cap stress is added to the model, the auxin increase at the non stressed side is augmented (Fig. 2A, bottom). Internalization of PIN2 from the apical membrane can hypothetically lead to elevated deposition of PIN2 on the lateral membrane. Indeed, our earlier data suggested a small increase in localization of PIN2 on the lateral inward membrane of cells at the salt-stressed side of the root (Galvan-Ampudia et al., 2013). Addition of this lateral upregulation in the extended model increases the auxin asymmetry (Fig. 2A, bottom; Fig. 2B, right), this was also the case when instead of a lateral upregulation, a basal upregulation of PIN2 was added (data not shown). However, when this same increase in lateral PIN2 was added to the baseline rectangular root model, the decrease in auxin on the non-salt-exposed side was even more severe (Fig. 2A, top). Thus, while lateral PIN proteins potentiate the translocation of auxin from the stressed to the non-stressed side in the realistic root tip architecture, in a rectangular root tip architecture this merely further cripples the effectiveness of the reflux loop.

Looking at the auxin rerouting map (Fig. 2C, right) for the wedge-shaped root, we observe a moderate rerouting of auxin to the lowermost parts of the non-salt-exposed side. Note, however, that for the wedge-shaped root, a threshold of 10% auxin increase is used for mapping auxin rerouting, whereas we used a 1% threshold value in the baseline square root model. Applying this lower threshold value in the wedge-shaped root model would reveal that auxin rerouting extends more shootward, consistent with the small increase in epidermal auxin levels (Fig. 2A, bottom; Fig. S1). The increase in auxin levels at the non-salt-exposed side of the root involves an initial rerouting against the normal direction of auxin transport as dictated by the polar PIN pattern of the reflux loop (Fig. 1A). This rerouting arises from auxin accumulating because of a lack of upward-oriented PIN2 transport, thereby increasing auxin uptake by the cells below it, now also causing accumulation in this cell, thus leading to the backward propagation of accumulated auxin. Only once the midline of the root is passed can this accumulated auxin join the normal direction of transport at the opposite non-salt-exposed side of the root. Our results suggest that a more realistic wedge-shaped root tip is essential for at least some of the accumulated auxin to reach this midline and become rerouted to the non-exposed side.

We hypothesized that the potential to re-route auxin to the non-exposed side critically depends on the shorter distance between left and right epidermis (and lateral root cap) tissue in the extended model compared with the baseline model. To test this, we use a variant of the extended model in which the root tip still has a wedge shape and contains a lateral root cap, but these distances have been increased (Fig. 1A, right). This results in a reduction of the auxin increase at the non-salt-exposed side of the root (Fig. 2A, bottom), thus confirming our hypothesis.

Note that the maximum observed increase in auxin levels at the non-stressed side of the root are 12-14% (extended model, salt-stress induced decrease of apical and increase of lateral PIN2 levels). This is substantially less than the auxin increase observed during halotropism experiments.

### AUX/LAX pattern and auxin feedback on its expression

**Fig. 3.**
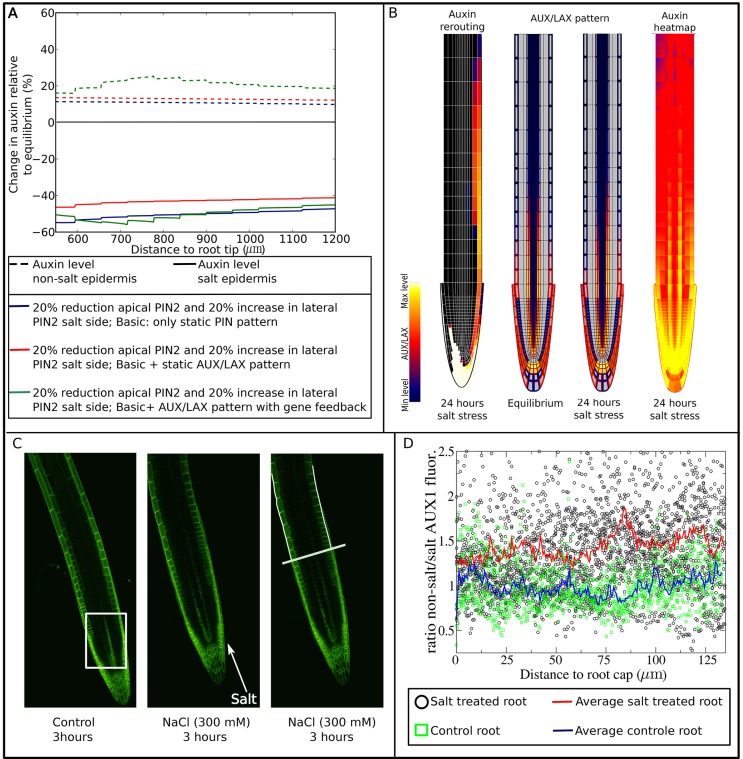
**Role of auxin-dependent AUX/LAX.** (A) Impact of AUX/LAX pattern and auxin feedback on AUX/LAX expression on epidermal auxin asymmetry 24 h after applying salt stress by reducing apical and increasing lateral PIN2 levels. (B) Auxin rerouting, change in AUX/LAX expression pattern and resulting auxin asymmetry in presence of auxin feedback on AUX/LAX expression. (C) AUX1-mVenus pattern in control root showing in the boxed region an asymmetric AUX1 fluorescence in the vasculature due to root orientation (left), and in a salt-exposed root showing generation of an asymmetric AUX1 pattern in the epidermis (middle). The right image shows a line transverse to the root length axis indicating the end of the lateral root cap, and two lines tracking the outer epidermal membranes in which AUX1 fluorescence levels are measured from the end of the root cap shootward. (D) Ratios of AUX1 fluorescence levels of the non-exposed to the salt-exposed side as a function of distance from the lateral root cap. Six roots were used for the control; for salt treatment, data from 12 roots were used. For details, see Materials and Methods.

Until now, influx of auxin from the walls into the cell was assumed to be equal for all cells. However, active import of auxin occurs by AUX/LAX membrane proteins, which exhibit a tissue-specific expression pattern (Bennett et al., 1996; Swarup et al., 2001, 2005; Péret et al., 2012). In gravitropism, AUX1 is essential for the adequate propagation of the initial auxin asymmetry (Bennett et al., 1996; Swarup et al., 2001). Therefore, we decided to investigate the potential role of AUX/LAX importers in halotropism. Focusing on the changes in auxin levels in the elongation zone of the root (≥500 μm from root tip), incorporation of the AUX/LAX tissue-specific expression results in an increase of auxin levels on both sides of the root (Fig. 3A). This logically follows from the preferred retention of auxin in AUX/LAX-expressing cells, such as the epidermis. Overall, auxin asymmetry actually decreased as a result of AUX/LAX incorporation into our model. However, the expression of auxin importers is known to be positively regulated by auxin (Laskowski et al., 2006, 2008). This auxin dependence may allow AUX/LAX importers to respond to and amplify auxin asymmetry. Earlier *in silico* studies have demonstrated the patterning potential of auxin feeding back on its own transporters (Runions et al., 2014). Addition of this auxin dependence indeed led to an increase in asymmetry, specifically the non-stressed side increased in auxin level (Fig. 3A), resulting from a substantially increased rerouting of auxin to this side (compare Fig. 2C, right, and Fig. 3B). Indeed, auxin levels at the non-salt-exposed side now increased by ∼30%, which is close to the factor 1.3-1.4 increase we estimated to occur during halotropic root bending. Another difference is that compared with Fig. 1B, middle, and Fig. 2B, right, the auxin pattern in Fig. 3B more closely resembles experimentally measured patterns with low auxin levels in meristematic epidermal cells, and epidermal auxin levels increasing above the end of the root cap where the elongation zone starts (Brunoud et al., 2012; Band et al., 2014) (see also Fig. S2). Clearly, incorporating realistic AUX/LAX patterns is crucial for correctly simulating auxin patterns. Underlying the enhanced halotropic auxin asymmetry, we see that AUX/LAX expression decreased on the salt-exposed side and increased on the non-exposed side (Fig. 3B). This asymmetry in AUX/LAX expression in not restricted to the epidermis, as the auxin importers are also asymmetrically expressed in the vasculature (Fig. 3B). This vascular asymmetry is consistent with the observed asymmetry in auxin signaling in the vasculature in halotropism experiments (Galvan-Ampudia et al., 2013).

Next, we set out to experimentally validate the asymmetry in AUX/LAX expression predicted by our model, focusing on AUX1 as the major auxin importer involved in tropisms. We assessed AUX1 membrane occupancy patterns, assuming that AUX1 membrane occupancy is linearly related to AUX1 expression levels, as is the case in our model. Fig. 3C shows an AUX1 pattern in a control root, which is expected to have a symmetric AUX1 pattern. Note the apparent asymmetry in vascular AUX1 patterns, which is due to the protophloem cells not always lying symmetrically in the focal plane. While this is something that can be avoided in many studies, this is not the case in our study where we do not want to interfere with root orientation relative to the salt gradient. For these reasons, we focus on epidermal AUX1 patterns for which both sides are clearly visible (Fig. 3C). As can be seen in Fig. 3B, our model predicts AUX1 patterns to be symmetric close to the root tip, and start diverging shootward from the rootcap. To test for such a spatial pattern, we measured AUX1 levels in the epidermal outer membranes, allowing us to assess the longitudinal AUX1 membrane pattern starting from the rootcap and going shootwards, and determine the development of left-right differences along this axis by computing ratios between non-exposed and salt-exposed sides (Fig. 3D). Consistent with our model predictions, close to the root cap, salt-exposed roots show an approximately symmetric AUX1 pattern comparable to that of control roots (ratio close to 1), whereas higher up, asymmetry builds up with the non-exposed side having higher AUX1 levels than the salt-exposed side (ratio close to 1.45). We tested for statistical significance of these findings by binning AUX1 ratios in 5000 µm spanning length segments for both salt-exposed and control roots, using a double-sided *t*-test to test per segment whether AUX1 ratios differ significantly. All segments were found to differ significantly (Table S1).

### Auxin feedback on PIN2 membrane levels

PIN proteins are constitutively cycling between the membrane and cytoplasmic vesicles. Experiments suggest that auxin may reduce the internalization of PINs, which would allow it to enhance its own export from the cell (Paciorek et al., 2005). However, effects are significantly stronger for synthetic than naturally occurring auxins (Paciorek et al., 2005; Rakusová et al., 2011). On the other hand, more indirect interactions involving SCF (TIR1/AFB)-auxin signaling and GOLVEN peptides, also appear to cause auxin-dependent regulation of PIN2 levels on the membrane (Baster et al., 2012; Whitford et al., 2012). When this feedback is added on top of the AUX/LAX pattern and feedback, the asymmetry in auxin is increased, especially in the lower part of the elongation zone, while auxin asymmetry in higher parts of the root decreased (Fig. S3A). Note that the concurrent asymmetry in PIN pattern underlying this (change in) auxin asymmetry is considerably smaller than the one observed for AUX/LAX, but again shows a decrease in the salt-exposed side and an increase on the non-exposed side (Fig. S3B).

### Salt-induced upregulation of PIN1

**Fig. 4.**
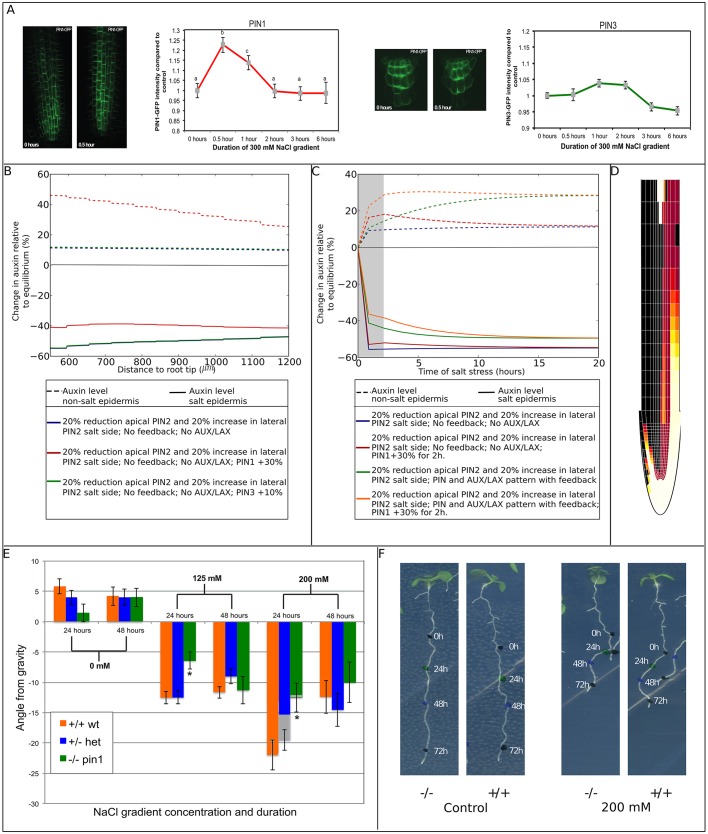
**Influence of salt gradient-induced changes in PIN1 and PIN3** **levels.** Representative images of roots of *A. thaliana* seedlings expressing PIN1-GFP (A, left) or PIN3-GFP (A, right) in control conditions (0 h) and after 30 min of a 300 mM NaCl gradient (0.5 h). Quantification of total PIN1-GFP and PIN3-GFP intensity at different time points in *A. thaliana* stele root cells is shown in the graphs. Three independent biological replicates were used in which 2-6 roots were used per time point and 5 stele cells were analyzed per root. For PIN1, *n*=90 for 0 h; *n*=25 for 0.5 h; *n*=55 for 1 h and 2 h; *n*=50 for 3 h and 6 h; for PIN3, *n*=70 for 0 h; *n*=20 for 0.5 h; *n*=60 for 1 h; *n*=55 for 2 h; *n*=40 for 3 h; *n*=35 for 6 h. Letters indicate different significance groups as determined by multivariate ANOVA. PIN1 levels show a 22% increase relative to control after 30 min of exposure to a salt gradient, after 1 h, this increase is reduced to 14%, and after 2 h the PIN1 level showed no significant difference relative to the control condition. PIN3 protein levels increase 4% compared with control conditions after 1 h of exposure, after 3 h, levels dropped to a 5% decrease compared with control conditions. (B) Impact of persistent salt-induced upregulation of PIN1 or PIN3 on epidermal auxin asymmetry after 24 h of salt stress. For comparison purposes, auxin asymmetry in the absence of PIN1 and PIN3 regulation is also shown. Owing to the lack of effect of PIN3 upregulation, control and PIN3 upregulation lines are superimposed. (C) Influence of transient PIN1 upregulation on the temporal dynamics of epidermal auxin asymmetry at a distance of 590 μm from the root tip. The 2 h period of PIN1 upregulation is indicated by the gray area. PIN1 upregulation is applied both in absence and presence of an AUX/LAX pattern and feedback of auxin on AUX/LAX expression and on PIN2 membrane occupancy. For comparison purposes, auxin asymmetry dynamics in the absence of transient PIN1 upregulation are also shown. (D) Auxin rerouting in the presence of transient PIN1 upregulation and auxin feedback on AUX/LAX expression and PIN2 membrane occupancy. (E) The *pin1* mutant shows a transient reduction in halotropism response after 24 h. Seeds from a heterozygous *pin1* parent were germinated on 0.5 MS plates and after 5 days exposed to a diagonal NaCl gradient. Seedlings that were homozygous for the *pin1* tDNA insertion (−/−) showed a reduced response to the NaCl gradient on both plates with a 125 mM and a 200 mM salt gradient compared with seedlings that were heterozygous (+/−) and seedlings homozygous for the wild-type (Col-0) allele (+/+). This reduction was observed after 24 h but not after 48 h. Two independent biological replicates were used in which 200 seedlings per treatment were quantified and genotyped. This resulted in +/−, *n*=105; +/+, *n*=56; −/−, *n*=44 for the 0 mM treatment. For the 125 mM treatment, the sample sizes were: +/−, *n*=122; +/+, *n*=120; −/−, *n*=59. For 200 mM, +/−, *n*=93; +/+, *n*=71; −/−, *n*=45. (F) Representative images of seedlings homozygous for the *pin1* wild-type allele (+/+) or seedlings homozygous for the tDNA insertion (−/−) on control plates or plates with a 200 mM NaCl gradient. Colored dots were placed at the root tip after 0 (1st black dot), 24 (green dot), 48 (blue dot) and 72 (2nd black dot) hours after applying the salt gradient. The angle from gravity after 24 h (the angle between the line from the first to the second dot and a line straight down) is significantly smaller in the example of the −/− seedling on a 200 mM NaCl gradient compared with the wild-type seedlings.

In an earlier study, we performed a control experiment aimed at verifying whether applied salt concentrations would affect internalization of PIN proteins other than PIN2. Cellular PIN1, PIN2, and PIN3 levels were determined under uniform salt exposure (Galvan-Ampudia et al., 2013). Intriguingly, PIN1 and PIN3 were found to be significantly upregulated by salt. Since these PIN1 and PIN3 elevations were observed under uniform high-level salt exposure and measured at a single time point, we now investigated to what extent physiologically relevant changes in PIN1 and PIN3 membrane levels arise in response to a salt gradient over the course of time (Fig. 4A, Fig. S4).

We find that, consistent with the earlier observed increase in cellular PIN1 levels, membrane levels of PIN1 increase by ∼22% (Fig. 4A left panels, Fig. S4A). However, the increase in PIN1 levels is transient. After 2 h of exposure, membrane levels of PIN1 had returned to its normal non-salt-stressed level (Fig. 4A, Fig. S4A). No indications for differences in PIN1 upregulation between the salt-exposed and non-exposed side of the root were found. Strikingly, in contrast to the earlier observed large increase in cellular PIN3 levels, membrane levels of PIN3 showed only a 4% transient upregulation (Fig. 4A, right panels, Fig. S4B). The observed salt-induced changes in PIN1 and PIN3 protein levels were similar for different membrane compartments as well as the intracellular compartment (Fig. S4), indicating that changes are not caused by changes in membrane-cycling dynamics. The difference in findings between current and earlier experiments is likely to be caused by the much higher concentration of salt applied when dipping the roots in uniform salt concentration, compared with the more subtle treatment of growing seedlings on a salt gradient (Fig. S4).

Returning to our computational model, we investigated how the transient increases in PIN1 and PIN3 could contribute to generate auxin asymmetry. First, the influence of 30% and 10% elevations in PIN1 and PIN3 levels, respectively, were investigated in isolation, ignoring for a moment auxin feedback on auxin transporters and the transient nature of the PIN1 and PIN3 elevations. Elevation of PIN1 levels significantly enhances auxin asymmetry, in particular by elevating auxin at the non-salt-exposed side (Fig. 4B). In contrast, the small elevation of PIN3 has no observable effect on auxin asymmetry. Therefore, we restrict our further analysis to PIN1. Next, we added the transient nature of the increase in PIN1 to the model, both in isolation and in a setting incorporating the feedback of auxin on auxin transporters. We plotted the changes in epidermal auxin levels at the start of the elongation zone (590 μm from the root tip) as a function of time (Fig. 4C). Interestingly, we see an elevated auxin asymmetry relative to control conditions during the transient PIN1 elevation in absence of feedback (Fig. 4C, red versus blue lines), indicating that symmetric PIN1 elevation contributes to generation of auxin asymmetry. This increased asymmetry gradually disappears once PIN1 has returned to normal levels (Fig. 4C), indicating that a transient PIN1 increase has no persistent effect on auxin asymmetry. If we combine the transient PIN1 elevation with feedback on auxin transporters, we see that increases in PIN1 cause an asymmetry that is slightly larger than that generated by feedback alone (Fig. 4C, compare orange and green lines), and that in the presence of feedback, the PIN1-induced auxin asymmetry decreases more slowly (Fig. 4C, compare orange and red lines). Most importantly, the transient PIN1 increase enhances the auxin asymmetry present during the first hours of halotropism (compare speed of auxin rerouting in Fig. 3B and Fig. 4D). In addition to speeding up the build-up of auxin asymmetry, a transient PIN1 elevation also causes an overshoot in auxin asymmetry levels during the early phases of halotropism. This agrees with our earlier observation that auxin asymmetry is higher after 4 h than after 6 h of salt exposure (Galvan-Ampudia et al., 2013).

To test the computationally predicted importance of PIN1, we tested halotropic responses on a salt gradient over a 48 h time course in plants homozygous for the *pin1* tDNA insertion compared with plants having a single or double copy of the wild-type *PIN1* allele. In Fig. 4E,F, we show the angle from gravity 24 h and 48 h after exposure to a 125 mM or 200 mM salt gradient or control conditions. For the 24 h time point, we see that for both salt concentrations, plant homozygous for the *pin1* tDNA insertion showed a significantly smaller angle from gravity relative to both heterozygotes and plants homozygous for the wild-type allele, indicative of a reduction of halotropic response strength in the absence of PIN1. After 48 h of exposure to a salt gradient, the homozygous *pin1* tDNA plants showed similar angles. This demonstrates that PIN1 conveys only a transient increase in halotropic response strength that is no longer present at 48 h, consistent with our model predictions. Note that the decrease in angle from gravity from 24 to 48 h can be understood from gravitropic signaling becoming stronger, thus counteracting the halotropic growth away from gravity, consistent with our earlier finding (Galvan-Ampudia et al., 2013). The stronger decrease under higher salt concentrations is likely to arise from the higher initially attained angles more strongly inducing gravitropism.

### Robustness of the results

In complex models such as these, testing the robustness of simulation outcome to specific model settings is of paramount importance to determine whether a general mechanism rather than an obscure, rare outcome has been found. To investigate the dependence on particular model assumptions, we varied a model assumption influencing the location of the main source of auxin in our model, which might potentially affect auxin patterns and fluxes and hence auxin asymmetry generated under halotropism. In the current model, all root cells have a small potential to produce auxin, and a major source of auxin is provided by influx from the shoot. However, recent data indicate that localized, root tip auxin production plays a major role in shaping the root's auxin pattern (Ljung et al., 2005; Stepanova et al., 2008). Therefore, as an alternative, we performed simulations in which shoot to root auxin flux was reduced by 50%, while auxin production was elevated 100-fold in the QC and in root cap cells, auxin production was increased 50-fold while decay was decreased by a factor of 2. Parameter settings were chosen such that for ease of comparison similar overall auxin content was achieved. Fig. S5 shows that this change in model setup results in a highly similar auxin asymmetry pattern compared with the default model settings. Thus, a shift in main auxin source from the shoot-root connection to the root tip does not impact our model outcome.

Next, to investigate the robustness of model outcome to parameter values, we varied most of our model parameters over a range of 50% decrease to 50% increase of their original values. In Fig. S6, we show the outcomes of our robustness analysis: for all tested parameters we observed limited quantitative variation in auxin asymmetry, while maintaining qualitatively similar outcomes. Based on these results, we conclude that model outcomes depend to a limited extent and in a smooth, linear fashion on parameter settings, thus implying the robustness of our model outcomes.

## DISCUSSION

We recently described halotropism as a new directional response of plants roots allowing them to grow away from salt (Galvan-Ampudia et al., 2013). In the current study, we used a detailed model of plant root auxin transport to investigate whether our earlier observations can account for halotropic root bending.

Our simulation study points to the crucial role of root tip architecture. We find that in a simplified rectangular root model, a reduction of PIN2 on the salt-exposed side merely results in the accumulation of auxin in the meristem. In contrast, in a realistic wedge-shaped tip architecture, the PIN2 decrease generates a small increase in auxin at the opposite root side. We showed that this potential to re-route auxin from the salt-exposed to the non-exposed side depends positively on the presence of a lateral root cap, the increase of lateral PIN proteins on the salt-exposed side, and a limited distance between epidermis and root cap of salt-exposed and non-exposed side. Together, this indicates that the potential for lateral transport of the auxin accumulating at the salt-exposed side is of crucial importance. In addition, we demonstrated an important role for positive feedback of auxin on its own transporters. Auxin-induced upregulation of AUX/LAX importers substantially elevated the auxin asymmetry generated by root tip architecture. The predicted asymmetry in AUX1 pattern resulting from this feedback was confirmed experimentally. Finally, we demonstrated that PIN1 is transiently upregulated under a salt gradient. While this transient change in PIN1 levels has no effect on long term auxin asymmetry, it significantly enhances the degree of auxin asymmetry during the early stages of salt stress. We speculate that generating auxin elevation at a faster rate is important to ensure root bending away from the salt before the tip of the root has started to grow into the salt-contaminated area. We experimentally validated this predicted role of PIN1 in halotropism.

In conclusion, our study shows that a decrease in PIN2 on the salt-exposed side can function as the primary generator of auxin asymmetry, but is not enough to generate a sufficiently large auxin asymmetry sufficiently fast. For this, the feedback of auxin on its own transporters and the transient salt-induced upregulation of PIN1 play a crucial role. Interestingly, in gravitropism, the *pin3* mutant is not agravitropic (Kleine-Vehn et al., 2010), starch mutants remain partly gravitropic (Caspar and Pickard, 1989), but both the *pin2* (Müller et al., 1998; Luschnig et al., 1998) and *aux1* (Bennett et al., 1996; Swarup et al., 2001) null mutants are agravitropic. Based on this it has been suggested that other, PIN3/7-independent mechanisms for gravitropism exist (Wolverton et al., 2002). Our study suggests that PIN2 may be a candidate for such a secondary asymmetry-generating mechanism, provided that gravitropism can somehow influence PIN2 directly.

Our study is an important step in unraveling the mechanistic basis of halotropism. It can be computed that in the experiments we performed here and earlier (Galvan-Ampudia et al., 2013), the differences in salt concentration at both sides of the root are in the order of 4-9.5%. Thus, future studies should be aimed at deciphering how such small asymmetries in auxin levels can become translated into a single-sided PIN2 response and how this might be related to the seemingly contradictory findings of an initial increase in PIN2 levels soon after the application of salt stress, as observed by Zwiewka et al. (2015) and the reduction in PIN2 levels after 6 h of salt stress, as we reported earlier (Galvan-Ampudia et al., 2013).

In addition, future studies should be aimed at deciphering the interplay between different tropisms. Interestingly, we found here that the auxin asymmetry generated during halotropism is substantially smaller than that during gravitropism. However, salt has been shown to suppress the gravitropism-induced degradation of PIN2 (Galvan-Ampudia et al., 2013) while enhancing the degradation of starch (Sun et al., 2008), explaining why halotropism can at least temporarily overcome gravitropism. In our earlier study, we demonstrated that at low salt concentrations halotropism is insufficiently strong to override gravitropism, while for higher salt concentrations, the eventual takeover of halotropic by gravitropic growth depends on salt concentrations (Galvan-Ampudia et al., 2013), suggesting a quantitative tug of war between the two tropisms. Given the important role of PIN2 and AUX1 in both gravitropism (Chen et al., 1998; Abas et al., 2006; Luschnig et al., 1998; Müller et al., 1998; Bennett et al., 1996; Swarup et al., 2001) and halotropism (this study), and the important role of PIN2 in phototropism (Wan et al., 2012), these proteins probably represent the signaling nexus at which different tropism pathways converge and signal integration occurs.

A final important question for future research is how tropisms can function in different developmental or environmental conditions, corresponding to different overall auxin levels. For robust tropic responses to occur, this might imply that tropisms generate and plant cells respond to relative rather than absolute changes in auxin levels – an issue that so far has not been investigated.

## MATERIALS AND METHODS

### Summary of the computational model

We use a spatially extended, grid-based model of the *Arabidopsis* root, similar to earlier models (Grieneisen et al., 2007; Laskowski et al., 2008; Mähönen et al., 2014). The model incorporates a root tissue architecture with subcellular resolution, cell type and developmental zone-specific spatial expression domains and polarity patterns for the auxin-exporting PIN proteins and the auxin-importing AUX/LAX genes, auxin transport within cells and cell walls and across membranes, and feedback of auxin levels on PIN membrane occupation as well as on AUX/LAX gene expression. The source code for the simulation models can be downloaded from http://bioinformatics.bio.uu.nl/khwjtuss/HaloRoot/.

### Baseline model

#### Tissue lay-out

We started with a highly simplified, rectangular root model, similar to that used in earlier modeling studies (Grieneisen et al., 2007; Laskowski et al., 2008; Mähönen et al., 2014). Root tissue was simulated with a spatial resolution of 2 µm on an 80×925 µm^2^ grid. Individual grid points correspond to cytoplasm, membrane or cell wall. We assume an average cell width of 8 µm, and simulate a total of eight columns of cells across the width of our 2D root model, incorporating from outermost to innermost epidermal (blue), cortical (green), endodermal (yellow) and vasculature cells (red). The vasculature is connected to the quiescent center (QC, gray), and the lowest part of the root represents the columella (cyan) (Fig. 1A, left panel).

Given that tissue growth occurs on a substantially longer timescale of days relative to the minute to hours timescale on which changes in PIN2, AUX1 and auxin patterns occur in response to salt stress, we ignored tissue growth in the current model. We incorporated a subdivision of the root into a meristematic (MZ), expansion (EZ) and differentiation zone (DZ) (Fig. 1A) containing cells with a height of 8 µm, 60 µm and 100 µm, respectively. Similar to previous modeling studies, we incorporated the PIN polarity patterns typical for each cell type and zone (Grieneisen et al., 2007; Laskowski et al., 2008; Mähönen et al., 2014) (Fig. 1A). Together, this results in a reverse fountain auxin flux pattern that generates an auxin maximum in the QC (Grieneisen et al., 2007).

#### Auxin dynamics

Auxin dynamics were modeled on a grid point level, in a manner similar to earlier studies (Mitchison, 1980; Grieneisen et al., 2007; Mähönen et al., 2014). For an intracellular grid point *i*,*j*, auxin dynamics are described as:
(1)
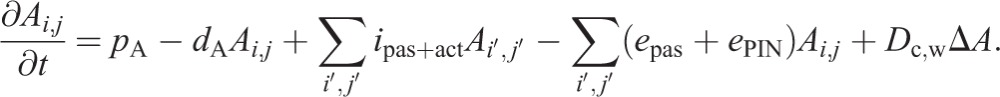
In Eqn 1 *p*_A_ is the rate of auxin production per grid point, and *d*_A_ the rate of auxin degradation per rate grid point, which are zero for wall points and non-zero for cellular grid points (membrane, cytoplasm). *i*_pas +act_ represents the lumped, active and passive import of auxin from all extracellular grid points *i*′,*j*′ that are neighboring the cellular grid point *i*, *j*, and *e*_pas_ and *e*_PIN_ correspond to the passive and active export of auxin from cellular grid point (*i*,*j*) to neighboring extracellular points (*i*′,*j*′), respectively. These transport reaction terms only exist for membrane and wall grid points. Auxin fluxes were modeled using linear mass action kinetics. Finally, *D*_c,w_ represents the diffusion constant for auxin inside the cell (c), or inside the wall (w) and diffusion occurs only among neighboring cellular or wall grid points, but not between cellular and wall points.

Note that *e*_PIN_=*a*_PIN_×PIN_mem_, i.e. rather than being a constant parameter, the rate of PIN-mediated active auxin transport depends on membrane PIN protein levels. Furthermore, PIN_mem_=PIN_pat_×PIN_exp_, that is, PIN membrane levels depend on the product of the PIN pre-pattern determining maximum relative PIN level at a particular membrane grid point (PIN_pat_) and the cellular PIN gene expression level (PIN_exp_) (Mähönen et al., 2014).

To model the connection of the explicitly modeled root section to the not-explicitly modeled rest of the plant we incorporate a shoot derived influx into the vasculature and efflux from the root to the shoot from the non-vasculature tissues, similar to earlier studies (Grieneisen et al., 2007; Mähönen et al., 2014). Parameter values are listed in Table 1.

**Table 1.**
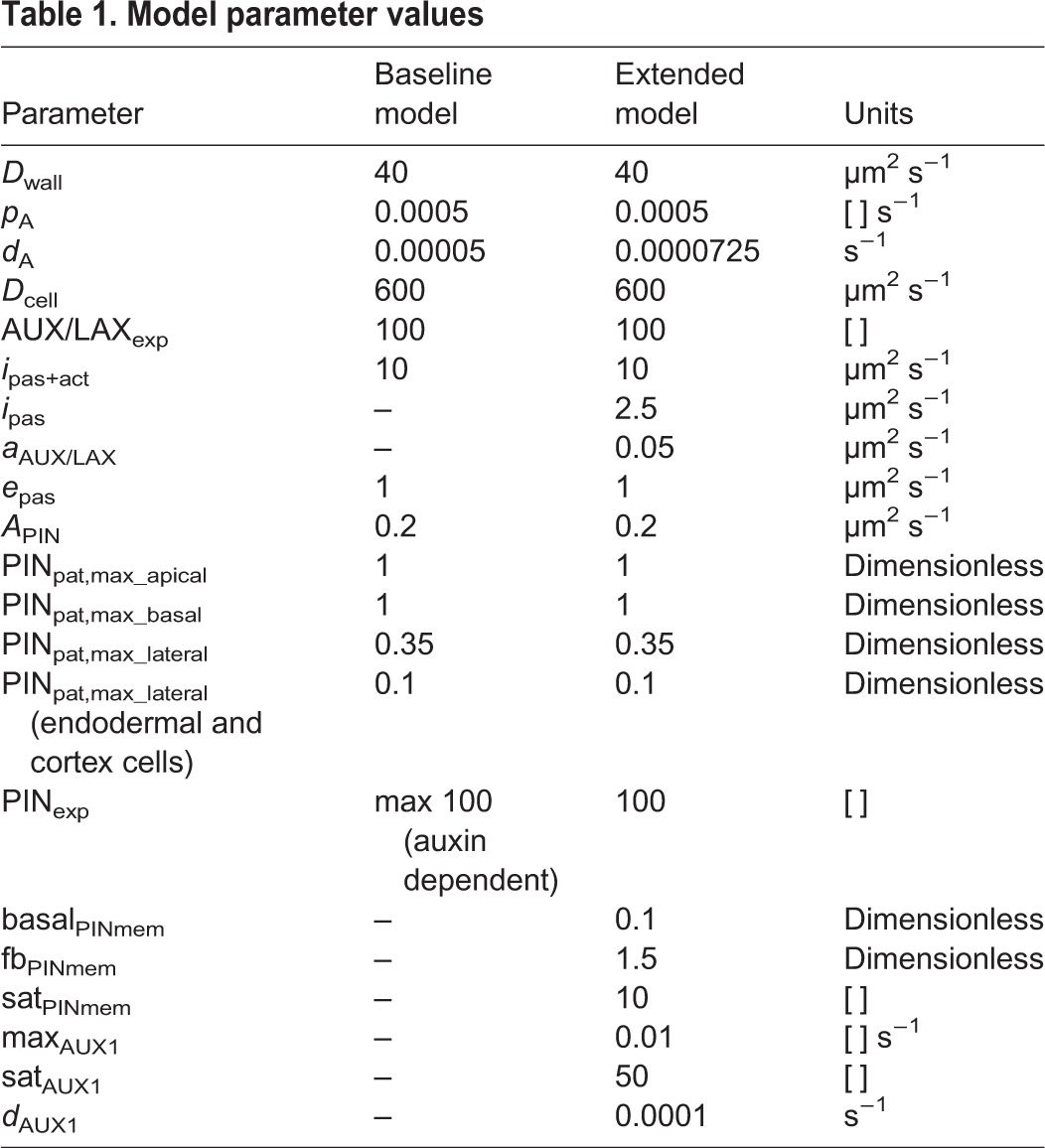
**Model parameter values**

### Extended model

#### Tissue lay-out

We also developed an extended model incorporating a more realistic, wedge-shaped root tip and root cap tissue (Fig. 1A, middle panel). The root tip template was generated by developing an idealized, perfectly symmetric, generalized description based on microscopic root tip pictures. The part of the root tip shootward of the lateral root cap was assumed to be perfectly straight and have constant width. In the part of the root containing the root cap, idealized parabolic functions were used to describe the outer boundary of the root cap, and the boundary between different root cap layers, between lateral root cap and epidermis, epidermis and cortex, cortex and endodermis, endodermis and pericycle, and pericycle and the rest of the vasculature. In the lower half of this part of the root, a central point was chosen, through which several radial lines were drawn that were used to describe cell walls in the columella, and lower parts of the lateral root cap, epidermis, ground tissue and vasculature.

Based on experimental data, root cap tissue has a similar PIN protein polarity pattern as epidermal tissue, but with lower maximum membrane PIN protein levels (Ditengou et al., 2008). In the extended model, cell types differ not only in PIN polarity patterns, but also have a cell type-specific width of 18 μm for epidermal, 20 μm for cortical, 12 μm for endodermal, 8 μm for the outermost vascular and 6 μm for the remaining vascular cells, in agreement with experimental data and similar to earlier models (Laskowski et al., 2008). Zone-dependent cell height was the same as in the baseline model, except for the curved part of the root tip where columella and lateral root cap cell height increases towards the tip of the root. In the extended model, to create sufficient resolution for the curved cell walls and membranes, a spatial resolution of 1 μm was used.

To test the effect of left-right distances between epidermis and lateral root cap on the potential to generate auxin asymmetries, we also developed a variation of the extended model in which these distances were increased (Fig. 1A, right). Increased distances between the epidermal tissues were generated by increasing the size of the outermost vascular cells to 10 μm and of the remaining vascular cells to 8 μm, increasing the distance between left and right epidermis with 16 μm. In addition, to also increase the distance between left and right epidermis and root cap in the tip of the root where cell files curve inward the leftmost and rightmost columella cells in each tier were increased in size.

#### AUX/LAX pre-pattern

In the baseline model, we assumed a constant, homogeneous distribution of auxin importers, and modeled this using a single lumped permeability value for passive and active auxin import *i*_pas +act_. However, it is well known that the AUX/LAX proteins involved in active auxin uptake have cell type- and root zone-specific patterns (Bennett et al., 1996; Swarup et al., 2001, 2005; Péret et al., 2012). Therefore, we distinguish passive and active auxin import in the extended model. For simplicity, we incorporated a single generalized AUX/LAX protein, whose domain of expression is the sum of the experimentally reported expression domains for the distinct AUX/LAX genes (Fig. S2). Similar to active efflux, the rate of active influx is then described by *i*_AUX/LAX_=*a*_AUX/LAX_×AUX/LAX_mem_, with AUX/LAX_mem_=AUX/LAX_pat_×AUX/LAX_exp_, where AUX/LAX_pat_ is the predefined spatial presence/absence pattern for where AUX/LAX can be expressed and AUX/LAX_exp_ is the actual cellular gene expression. In addition to this spatially heterogeneous active influx we also incorporate a constant, low level of passive auxin influx, *i*_pas_, in all cells. Parameter values are listed in Table 1.

#### Auxin-dependent gene expression of AUX/LAX

In a subset of simulations, we incorporate the auxin dependence of AUX/LAX gene expression (Laskowski et al., 2006, 2008). We do this by replacing constant AUX/LAX expression levels (AUX/LAX_exp_) by the following cell-level equation for gene expression dynamics:
(2)
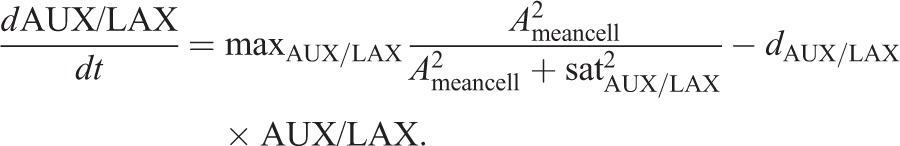
Here, max_AUX/LAX_ is the maximum gene expression rate of AUX/LAX, sat_AUX/LAX_ is the auxin level at which AUX/LAX expression is at its half maximum rate and *d*_AUX/LAX_ is the degradation rate of AUX/LAX. Parameter values can be found in Table 1.

#### Auxin feedback on PIN localization

PIN levels on the membrane not only depend on PIN gene expression levels but also on PIN membrane cycling dynamics. PIN proteins are constantly recycled by internalization from the membrane and subsequent secretion to the membrane (Steinmann et al., 1999; Adamowski and Friml, 2015). Auxin influences this subcellular trafficking by limiting the rate of internalization, thus stimulating its own efflux from the cell (Paciorek et al., 2005). This results in a positive feedback between external auxin and membrane PIN levels.

In a subset of simulations, we incorporated this positive feedback on levels of PIN in the membrane. We restricted this positive feedback to the epidermal and root cap tissues that are considered of primary importance for generating the auxin asymmetry underlying root bending. For these cells, the PIN membrane equation (PIN_mem_=PIN_pat_×PIN_exp_) is replaced by the following grid-level equation:
(3)
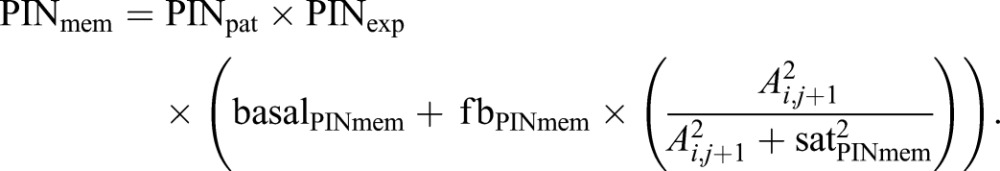


Here basal_PINmem_ is the minimal fraction of PINs on the membrane in the absence of auxin, and fb_PINmem_ is the maximal additional fraction of PINs on the membrane in presence of high levels of auxin. sat_PINmem_ is the auxin level at which this auxin-dependent fraction attains its half maximum value. Parameter values can be found in Table 1.

### Simulating halotropism

Currently, no quantitative data is available showing how changes in PIN2 levels depend on the longitudinal position of a root cell (i.e. distance to the root tip). For simplicity, we therefore assume the same constant response to salt along the first third of the simulated root tissue, while above this part, no response to salt is assumed to occur (Fig. 1A). The salt gradient was assumed to be localized to the left of the root tip. We considered two different halotropism scenarios. In the first, apical PIN2 levels were decreased by 20% in the epidermis and (if present) root cap at the salt-exposed side of the root. In the second scenario, concomitant with a reduction of apical PIN2 levels, a 20% upregulation of lateral PIN2 levels was assumed to occur. Both scenarios were based on the experimental data from Galvan-Ampudia et al. (2013). Simulations were run without salt stress until auxin concentrations reached an equilibrium, after which salt stress was applied.

### Analysis methods

Auxin levels may vary both because of the imposed salt gradient as well as due to different model settings. To faithfully compare the extent of auxin asymmetry resulting from a salt gradient under different model settings, we compare auxin levels in the left and right epidermis under salt stress with auxin levels under normal, non-stressed conditions with the same model settings. Furthermore, we compute percentage rather than absolute differences relative to these normal auxin levels. Formally, this can be written as:
(4)
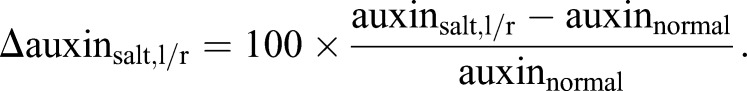


### Numerical integration and run-time performance

Owing to the very fast auxin dynamics, stable integration using simple forward Euler schemes would require very small temporal integration steps (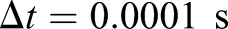) making simulations prohibitively slow. Therefore, a semi-implicit alternating direction integration scheme (Peaceman and Rachford, 1955) was used that allows for integration time steps of 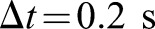. This approach has been extensively validated in earlier studies (Grieneisen et al., 2007; Mähönen et al., 2014).

All simulations were run on a dell Precision T7500 workstation with Intel Xeon X5680 processor. The code for the model was written in C++. Run time for simulations were typically around 24 h (corresponding to a biological time of a few days) to reach steady state gene expression and auxin levels in absence of salt stress, and 3-6 h for simulating salt stress (biological time of 10-36 h).

### Experiments

#### Growth conditions and treatments

We used *Arabidopsis thaliana* PIN1-GFP (Benková et al., 2003), PIN3-GFP (Zádníková, 2010), *pin1* (SALK_047613) and AUX1-mVenus (Band et al., 2014) lines, all in the Col-0 background. Seeds were sterilized using a 50% bleach solution. After 2 days of stratification the seeds were germinated on 0.5 MS plates with 0.1% MES buffer, 1% sucrose, 1% Daishin agar after which the pH was adjusted to 5.8 (using KOH). The plates were placed at an angle of 70° and placed in a climate chamber (22°C at long-day conditions, 16 h of light at 130 µmol/m^2^/s). After 4 days, the plants were transferred to new 0.5 MS plates. On day 5, the treatment was started. Salt gradients were created starting at 0.5 cm from the root tip by cutting the left lower corner of the square 0.5 MS plate and replacing this with fresh 0.5 MS medium containing 125 mM, 200 mM or 300 mM NaCl, depending on the experiment (for control plates medium was replaced with fresh 0.5 MS medium without salt). The plates were dried for 15 min and placed back into the climate chamber. Microscopy slides were prepared by cutting a rectangle around the seedling and placing it upside down on a microscopy slide while maintaining an angle of 70°. The slides were imaged within 5 min of removing the plates from the climate chamber.

#### Confocal laser-scanning microscopy

The images were acquired using a Nikon Ti inverted microscope in combination with an A1 spectral confocal scanning head. For GFP, the excitation wavelength used was 488 nm, the emission wavelength detected was 505-555 nm. For mVenus (YFP), the excitation wavelength used was 514 nm and the emission wavelength used was 525-555 nm. The analysis of the images was performed using Fiji (http://fiji.sc) software.

### Analysis

#### PIN1 and PIN3 response to salt gradient

Plants were exposed to a 300 mM NaCl gradient and imaged at different time points (0.5, 1, 2, 3 and 6 h). For control plants, no significant differences between time points were found. Therefore, all control plants could be pooled into a single control group. Three biological replicates were performed for both PIN1 and PIN3, each time with newly grown *A. thaliana* seedlings. In each experiment, six roots were imaged for the control condition, two roots for the 0.5 h of salt gradient and four roots for the other treatments (1 h, 2 h, 3 h and 6 h). Five cells from each root were used for the quantification of PIN membrane levels. We dismissed images in which an insufficient number of cells could be used for quantifying PIN membrane levels. This could be due to an unfortunate root angle, bad confocal plane or air bubbles near the root while imaging. Five cells of each imaged root were used to determine the average GFP intensity of the pixels by drawing a region of interest (ROI) around one side of the membrane or the intracellular part of the cell and using Fiji software to calculate the average. These values were then corrected for background fluorescence by subtracting the average value of a part of the root which does not express the specific PIN protein. Significance levels between control and salt conditions and the different time points were tested by using ANOVA (using SPSS software).

#### AUX1 response to salt gradient

Plants were imaged 3 h after exposure to a 300 mM salt gradient or control conditions. Three biological replicates were performed for the salt gradient and for control conditions. For the salt gradient a total of 13 plants were analyzed (5, 4 and 4 for the individual replicates), for control conditions a total of 9 plants were analyzed (4, 3 and 2 for the individual replicates). For each root, a line transverse to the longitudinal axis of the root was drawn to indicate the position at which the lateral root cap ends. Next, two lines were drawn following the outer lateral membranes of the epidermal cells starting from the end of the rootcap in the shootward direction (see Fig. 3C, right). YFP intensity levels of the pixels composing these lines were determined using ImageJ software (https://imagej.nih.gov/ij/). For each root, we computed the ratios between left and right epidermal AUX1 levels as a function of distance from the lateral root cap by determining the ratio of YFP intensity levels for pixel pairs consisting of a pixel at the left and a pixel at the right outer epidermal membrane located at the same distance from the end of the lateral root cap. For the salt treatment, one root was discarded from our analysis, for the control conditions, two roots were discarded from our analysis because of bad confocal planes resulting in highly uneven fluorescence levels at the left and right sides of the root prohibiting the proper application of the above explained analysis method. In addition, for control conditions, one root was discarded from our analysis because of the high level of root bending observed. It has been previously shown that root bending may induce local elevation of AUX1 levels (Laskowski et al., 2008). Ratios computed for salt gradient and control roots were binned per 5 µm length segments. Significance levels between control and salt gradient exposed roots were computed per bin using a double sided *t*-test (using R software).

#### Effect of *pin1* on halotropism

Seeds from a heterozygous *pin1* mutants (SALK_047613) [the homozygous *pin1* mutant is sterile (Okada et al., 1991)] were plated and grown to 5 days, as described above (growth conditions and treatment). A difference relative to the above described experiments is that the plants were not transferred after 4 days to new 0.5 MS plates but were germinated on the final treatment plates. On day 5, the treatment was started, as described above. The 5-day-old seedlings were then analyzed for their halotropic response according to Galvan-Ampudia et al. (2013). Ten seeds were used per plate and 20 plates for each treatment (0, 125 and 200 mM NaCl). All seedlings were genotyped to identify seedlings homozygous for the tDNA insertion [forward genomic primer: acggtatagtccctctataact, reverse genomic primer: gctgcaaaagagtgacataaa and insertion primer (LBb1.3): attttgccgatttcggaac]. Significance levels between genotypes at different time points were tested with SPSS software by using MANOVA (post hoc Bonferroni *P*<0.01).
